# Religiosity: A resilience factor protecting against depression in medical interns

**DOI:** 10.1016/j.actpsy.2025.105352

**Published:** 2025-08-04

**Authors:** April R. Kriebel, Yu Fang, Sanjay Saint, Srijan Sen, Margit Burmeister

**Affiliations:** a Department of Computational Medicine & Bioinformatics, University of Michigan, Ann Arbor, MI, USA; b Michigan Neuroscience Institute, University of Michigan, Ann Arbor, MI, USA; c Medicine Service, Veterans Affairs Ann Arbor Healthcare System, and Department of Internal Medicine, University of Michigan, Ann Arbor, MI, USA; d Department of Psychiatry, University of Michigan, Ann Arbor, MI, USA; e Department of Human Genetics, University of Michigan, Ann Arbor, MI, USA

**Keywords:** Medical interns, Depression, Religious attendance, Resilience

## Abstract

**Introduction:**

Religion has been proposed as a potential resilience factor against depression, but cross-sectional study designs and the lack of a universal stressor have impeded establishing a causative, longitudinal relationship between religious attendance and resilience to depression.

**Methods:**

Utilizing the national, prospective cohort Intern Health Study, we examined 5482 physicians as they entered the first year of residency in the following clinical disciplines: internal medicine, general surgery, pediatrics, obstetrics, gynecology, and psychiatry. Depression symptoms were quantified by the 9-item Patient Health Questionnaire, PHQ-9, scores. Religious attendance was self-reported on a scale ranging from 1- “Never” to 6- “More than once per week”.

**Results:**

We found that religious attendance prospectively predicted significantly lower depressive symptom development among interns. Among religious interns, more frequent religious attendance was associated with a smaller increase in depression symptoms after the start of internship. Specifically, with each level of increase in the frequency of religious attendance, the increase in PHQ-9 score was mitigated by an effect size of −0.10 Furthermore, the effect of religious attendance on PHQ-9 score was consistent across most ethnic groups, including Latinos, East Asians, Whites, African Americans, and Arabs.

**Conclusion:**

Religious attendance provides a dose-dependent protective effect against stress-induced depression throughout medical internship across many ethnic groups.

## 1. Introduction

There has been substantial interest in understanding whether religiosity and religious attendance offers tangible benefits to the well-being of individuals. Previous work has highlighted an association between an individual’s personal religious practices and mental health benefits, including the potential for resilience against depression (Baetz et al., 2004; Braam & Koenig, 2019; Brown, 2003; Levin, 2010; Maselko et al., 2009; Ronneberg et al., 2016). In a recent review (Brown, 2003), religiousness and spirituality were found associated with reduced depression in the majority of prior studies, but the “huge” heterogeneity in studies and sparsity of prospective studies led the authors to classify their conclusions as tentative and exploratory. Most of the studies performed to date have been cross-sectional in nature and thus not designed to assess whether religious practice predicts—and is causally related to—future depression, and the few prospective studies were often conducted in geriatric populations (Ronneberg et al., 2016). Previous studies suffer from a lack of a common, universal stressor, with participants reporting a wide range of stressful experiences that are difficult to analyze collectively or to quantify (Cheung et al., 2017; Koenig et al., 1992). To comprehensively assess religious attendance as a protective factor against depression, it is necessary to examine whether it serves as a buffer at the onset of a depressogenic situation, if it has a continual impact for the duration of the episode, and whether these effects are mitigated by stress.

Medical internship, the first year of medical residency after finishing medical school, is a period defined by long work hours, reduced sleep, and unique ethical dilemmas. Meta-analyses find that medical interns in the USA experience a 4–5 fold increase in depression with the onset of residency training (Mata et al., 2015). Previous investigations of religious attendance as a resilience factor in intern populations have been limited by their cross-sectional nature (Smith et al., 2003; Vasegh & Mohammadi, 2007), making it difficult to understand how religious attendance may predict future depression after the onset of stress.

Here, we use a prospective Intern Health Study (IHS) study to better understand whether religious attendance may serve as a resilience factor both at the initialization of the stressor (i.e., beginning internship), as well as throughout a stressful period (i.e., internship year). As religious attendance and depression rates have been found to vary by race and ethnicity, we also investigated each of the major races/ethnic groups separately to avoid confounding by racial or ethnic backgrounds. Religious attendance was self-reported on a scale ranging from 1 - “Never” to 6 - “more than once a week”, allowing a quantitative analysis of dose. A key innovation of our study is that the religiosity measure is gathered before the onset of stress — and the potentially subsequent increased depression — thereby making it possible to directly analyze the potential effects of religious attendance on resilience against depressive symptoms, without being confounded by selective bias or recall. We hypothesized that religious attendance before the stress onset is a significant and dose-dependent resilience factor of depressive symptoms development under stress (measured by the mean PHQ-9 score change from baseline during internship), while this effect could not be observed before the stress onset in baseline PHQ-9.

## 2. Materials & methods

### 2.1. Study population

The Intern Health Study (PI - Srijan Sen) is an annual cohort study of stress and depression of first-year medical residents (interns) in the United States. Prior to the start of the 2017–2018, 2018–2019, and 2019–2020 medical internship years, a total of 12,068 incoming interns from 39 geographically diverse recruiting institutions in the United States were contacted via email and asked to participate in the IHS. Participants provided informed consent online and were asked to complete a baseline survey upon enrollment and quarterly follow-up surveys at months three, six, nine and twelve of the internship year. Participants received between $50 and $125 in compensation, depending on the cohort year. The study, HUM00033029, was approved by the institutional review board at the University of Michigan on 12/26/2016, 10/15/2018, and 9/5/2019 for the relevant years. We combined the data from these three cohorts of interns for the subsequent data analysis. Of the potential enrollees, 6658 consented to participate in the study, for a participation rate of 55.2 %.

### 2.2. Measures

The baseline survey collected self-reported measures of the frequency of religious attendance as the measure of the interns’ religiosity. Religious attendance was assessed at baseline on a 6-point scale, with 1- “Never”, 2- “Once a year or less”, 3- “A few times a year”, 4- “A few times a month”, 5- “Once a week”, 6- “More than once a week”. The baseline survey also included basic demographic information (age, sex, self-reported ethnicity), prior personal history of depression, early family environment information, neuroticism, and a baseline depression score. The early family environment was assessed with the Risky Families Questionnaire (Taylor et al., 2006), which evaluates the family environment during childhood and early adolescence (age 5 to 15 years). A total of 13 questions assesses the frequency of 10 childhood experiences, such as insults, drinking, quarreling, and 3 positive events, such as loving and hugging, resulting in a scale of 0 to 65 (higher scores indicate a more chaotic, harsh, and abusive early family environment). Neuroticism was measured through the neuroticism subscale of Neuroticism, Extraversion, and Openness to Experience (NEO) Five Factor Inventory (Costa Jr & McCrae, 1997), which is a 14-item scale assessing negative emotional constructs, such as anxiety, mood, worry, envy, and jealousy, resulting in a total score of 0 to 56 (higher scores on the neuroticism subscale indicate an increased likelihood of experiencing negative emotions, especially in response to environmental stress). The depression score was measured with the 9-item Patient Health Questionnaire (Spitzer et al., 1999) (PHQ–9). The PHQ–9 score asks participants how frequently they had experienced specific depressive symptoms in the past two weeks, with responses varying from “0 = Not at all” to “3 = Nearly every day”. A PHQ-9 score equal to or greater than 10 has moderate sensitivity and specificity for a DSM diagnosis of major depressive disorder (Kroenke & Spitzer, 2002; Spitzer et al., 1999). In addition to baseline, PHQ–9 was also measured quarterly (at 3, 6, 9 and 12 months) during the intern year. Sleep hours (Kalmbach et al., 2017), work hours, the presence or absence of stressful life events and perceived medical errors were also assessed quarterly during the intern year (Sen et al., 2010). The stressful life events question asks: “During the past 3 months, have you experienced any of the non–internship-related stressful life events: death of family or friends, being ill or injured, ending of relationship, being in a violent relationship, financial loss or debt, being assaulted or attacked, getting married, the pregnancy or birth of a child?”

### 2.3. Statistical analysis

To assess if religious attendance served as a protective factor against a rise in PHQ–9, we constructed an ordinary-least-squares linear regression model that utilized religious attendance as the independent variable and mean PHQ–9 score change—defined as the mean PHQ–9 score over internship minus the individual’s baseline PHQ–9 score—as the dependent variable (See Eq. (1)).

(1) $$\text{Δ}PHQ-9=\frac{\sum_{i=1}^{4}PHQ-9_{Qi}}{4}-PHQ-9_{Baseline}$$

Because there is no significant difference in PHQ–9 score across quarters, we utilized mean PHQ-9 change across quarters as our primary outcome measure (Fang et al., 2022). Religious attendance, from 1 to 6 was used as a continuous predictor variable (Robitzsch, 2020). We first created the linear regression model with no control variables. Next, to assess what control variables should be included into the model, we iteratively included factors previously associated with PHQ–9 score change: prior depression history, sex, baseline neuroticism scores, early family environment scores, work hours, stressful life events, perceived medical errors, normalized baseline PHQ–9 scores, cohort year, and average hours slept in the model, and retained them only if they improved the adjusted *R*^2^ of model fit (Ge et al., 2017; Sen et al., 2010).

We then examined whether a particular sex or ethnicity was more inclined to religious attendance within our population, and whether the effect of religiosity to reduce depressive symptoms differs by sex or ethnicity groups. We assessed potential differences using a Welch’s *t*-test to compare religious attendance between males and females, as well as between ethnicities, using Whites as the reference group. Interns not categorized into a main ethnic group (i.e. White, Latino, African American, Arab, Southeast Asian, East Asian) were denoted as “Other”. We calculated effect size using Cohen’s *d*.

Further, we included an interaction term between religious attendance and ethnicity, using Whites as our reference population. For this model, we constrained our population to exclude those participants who identified as multi-racial, other, or NA, and those who identified as Native American, due to small sample size (*N* = 5). In addition to the interaction term between religious attendance and ethnicity, we include all the same control variables. We also constructed a third model by adding an interaction term between religious attendance and sex.

We also conducted a secondary analysis investigating the relationship between religious attendance and PHQ-9 scores at baseline. We first normalized the distribution of the baseline PHQ–9 scores. We then constructed a linear model such that the independent variable was religious attendance, and the dependent variable was baseline PHQ–9 score. We evaluated whether prior depression, baseline neuroticism scores, sex, early family environment scores, cohort year, and stressful life events at baseline would be appropriate control variables by iteratively adding them to the linear model. If the control variable improved the adjusted *R*^*2*^ of model fit, the control variable was retained.

All statistical analysis was performed in R (v4.0.0.) with packages ‘lme4’ (v1.1.27) and ‘lmerTest’ (v3.1.3).

#### Data Access:

All Intern Health Study data are available in the open Inter-university Consortium for Political and Social Research repository: https://www.openicpsr.org/.

## 3. Results

### 3.1. Basic demographics and depression onset is within normal ranges for the assessed cohorts

After excluding participants who did not report the assessed control variables, we included 5482 subjects in study analyses. The demographics of our sample are outlined in Table 1.

**Fig. 1.**
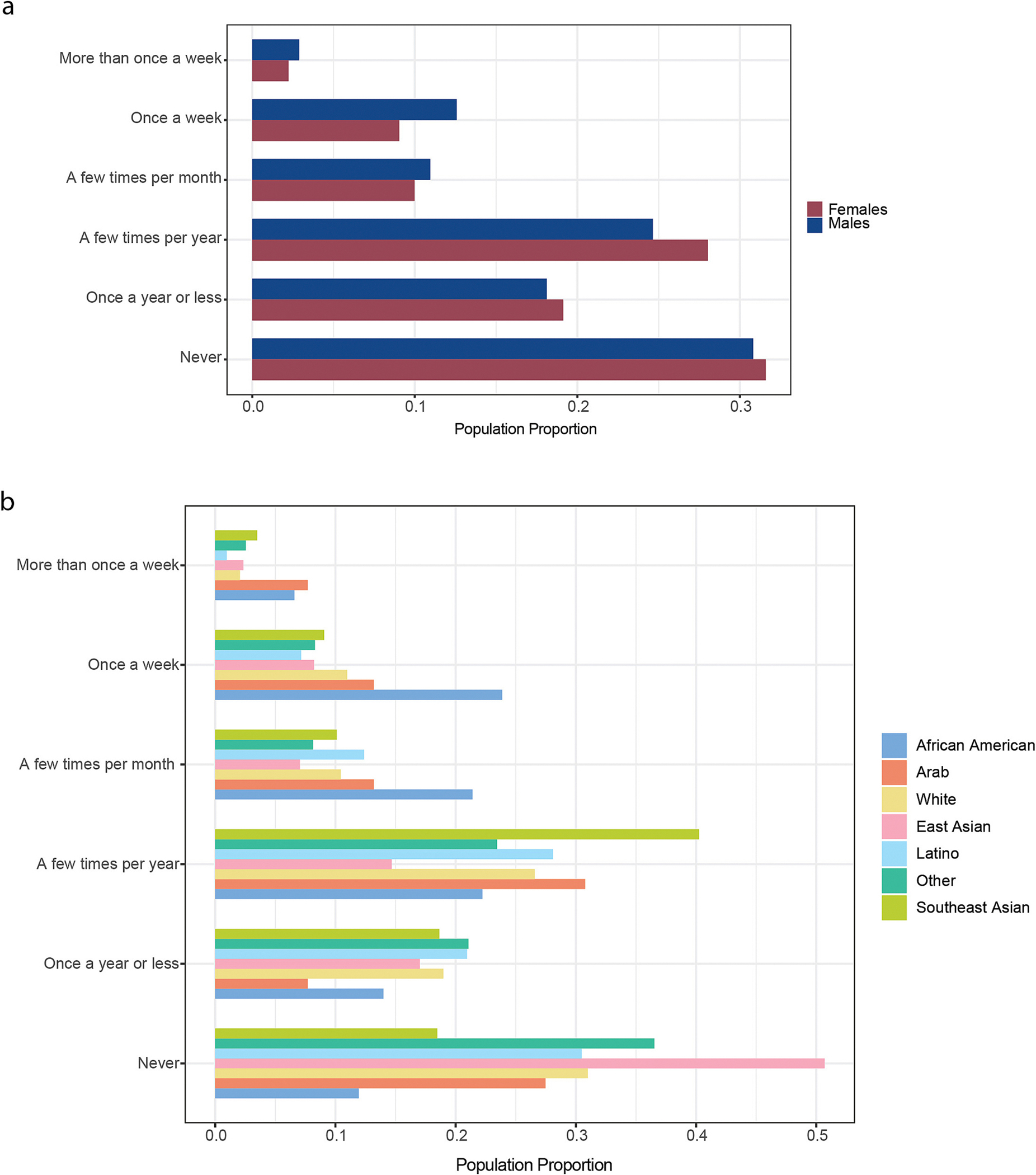
The population proportions for religious attendance differed by sex and ethnicity. Comparing the general population proportions between sex (a) and between ethnicities (b) for each religious attendance category demonstrates variance across both sex and ethnicity.

Overall, 31.2 % of interns reported never attending a religious service while 23.6 % reported the highest level of religious attendance (a few times per month or more frequently). In our sample, we found men to be more religious than women (*p* = 0.001, Welch’s Test) (Fig. 1a, Table 2). There were also significant differences in religious attendance practices between ethnicities (Fig. 1b, Table 2), where African Americans were found to be more likely, and those of East Asian ancestry less likely than the reference group, Whites, to attend religious services. Before internship began, 3.8 % of medical interns met PHQ criteria for depression. Cumulatively, 34.4 % of medical interns met PHQ criteria for depression (Kroenke & Spitzer, 2002) at least once during the internship year.

The mean increase in PHQ–9 score from baseline (2.6) to internship (5.7) for our cohorts was consistent with previous studies findings (10) (*t* (5481) = [−69.52], *p* < 0.001, *d* = 0.95, paired t-test).

### 3.2. Religious attendance demonstrates a dosage-effect mechanism

**Fig. 2.**
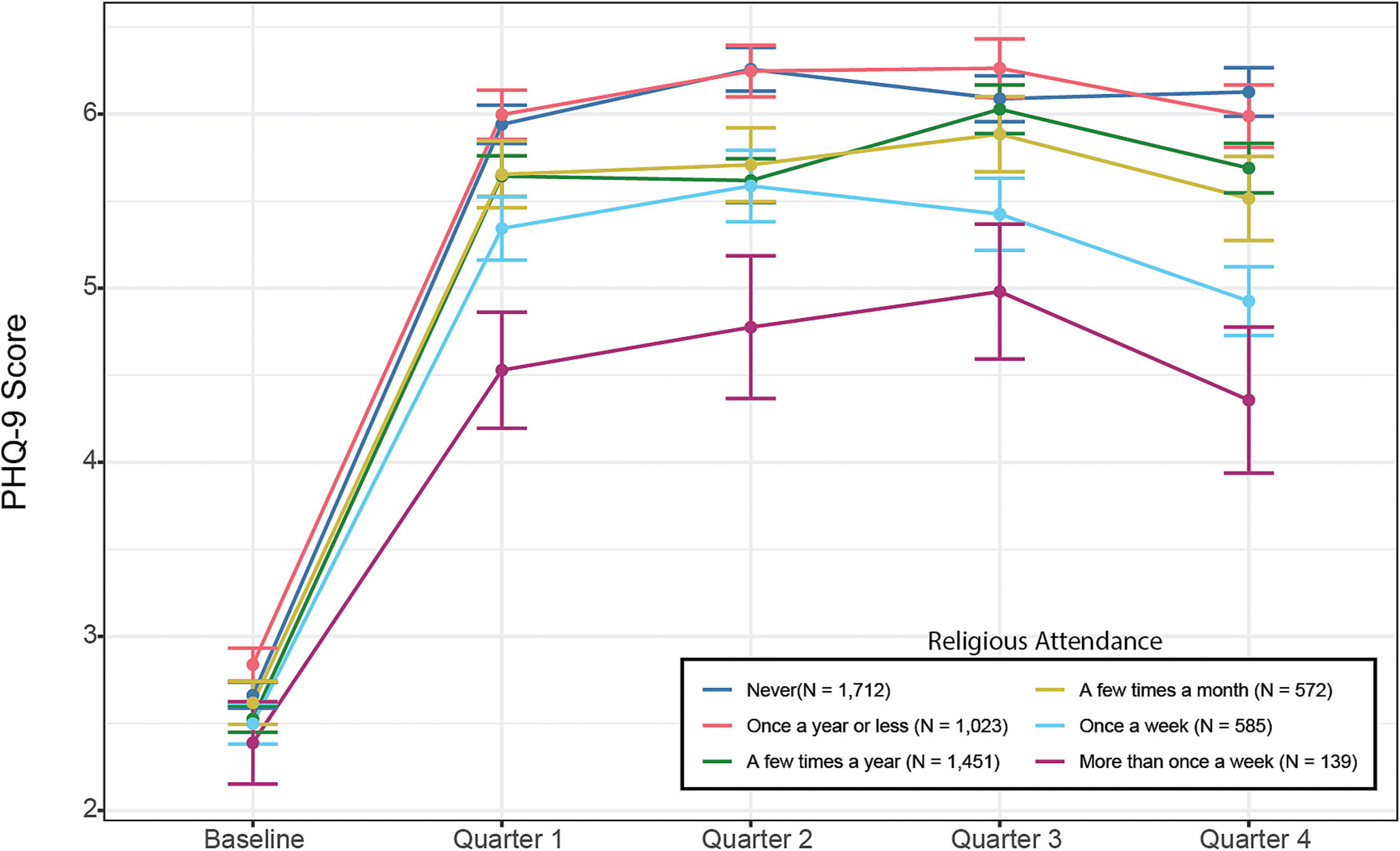
The relationship between religious attendance and the development of depression symptoms. The error bars represent standard error.

After covariates selection, we used prior depression, sex, baseline neuroticism scores, work hours, perceived medical errors, baseline PHQ–9 scores, cohort year, and average hours slept as control variables. Before (data not shown) and after adjusting for control variables, more frequent religious attendance was significantly associated with a smaller overall increase in PHQ–9 score (*β* = −0.10, *p* = 0.002) (Fig. 2, Table 3). Those never attending religious services experienced the largest increase in depressive symptoms with stress (M = 3.5, SD = 3.7), with a decrease in mean PHQ–9 score change with each stepwise increase in religious attendance frequency (Table 4). Baseline PHQ–9 score was not significantly associated with religious attendance before (Data not shown) or after adjusted for covariates (*β* = 0.01, *p* = 0.322) (Table 3).

**Fig. 3.**
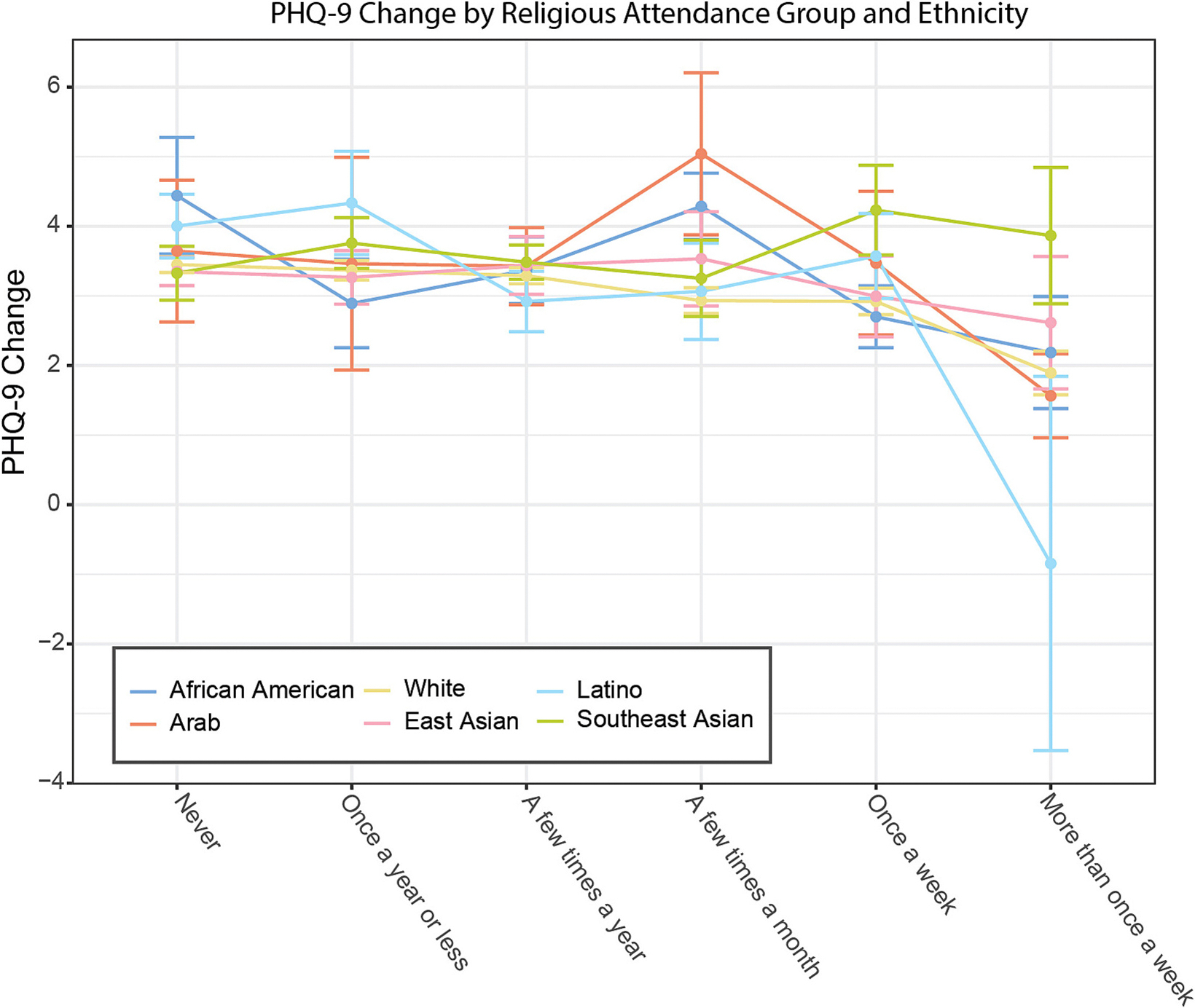
The relationship between religious attendance and the development of depression symptoms, by ethnic groups. For each self-reported ethnicity, we calculated the mean PHQ-9 score change that occurred over the duration of internship for each religious attendance group. Error bars represent standard error.

The interactions between religious attendance and ethnicity groups (*N* = 4855) were not significant for those who self-identified as African American, Arabic, East Asian, or Latino, using the White population as the reference population. For those who identified as Southeast or South (SE/S) Asian, there was a nominally significant interaction (*p* = 0.039; *β* = 0.358) with religious attendance (Fig. 3). There was no significant interaction between sex and religious attendance when predicting change in PHQ–9 score (*p* = 0.19, data not shown).

## 4. Discussion

In this study, we utilized a national, geographically diverse, and prospective Intern Health Study to investigate whether religious attendance may serve as a resilience factor throughout a stressful period. We found that more frequent religious attendance at baseline was associated with a significantly lower mean depressive symptom score increase during the internship year, supporting the notion that religious attendance can serve as a resilience factor among people under stress. The dose-response relationship between religious attendance and depressive symptoms is evident from the mean change in PHQ–9 score decreasing with each successive increase in religious attendance frequency (Fig. 1). More frequent religious attendance could indicate a firmer conviction in the tenants being practiced and might be protective because of the religious practice itself. Religious attendance could also provide a measure of resilience by providing an individual with a consistent sense of community, a previously identified source of resilience (Ge et al., 2017).

The significant relationship between more frequent religious attendance and a reduced increase in depressive symptoms had a small effect size. While this indicates that more frequent religious attendance influences interns’ resilience, it also highlights the complexity underlying depression and depression-like symptoms. The small effect size of religious attendance indicates that other factors must contribute to resilience. Given the complicated nature of mental health, a small effect size is expected.

Higher religiosity has been correlated with higher grade point average, lower truancy rates, and higher levels of academic attainment (Horwitz, 2021). It has been suggested that the ethics instilled in religious youths promotes an attitude of compliance and respect for authority, which in turn results in better school performance. Increased social capital has also been cited as a key driver in the academic success of religious youths (Horwitz, 2021). Yet, another possibility must now be considered and explored. Namely, when faced with stress-inducing situations, are more religious students more likely to be more resilient to depressive-symptoms and maintain adequate scholastic performance? The interaction between resilience and religiosity may not be constrained to only paradigms of severe stress, and this relationship should be further explored.

By examining the effect of religious attendance on depression symptoms during the duration of internship, we were able to better assess the manner in which religious attendance may operate as a resilience factor. The presence of a consistent difference in depression symptoms scores across the entirety of the one-year internship suggests that religious attendance is able to function as a protective mechanism both at the initial onset of a stressor, as well as in the protection against a longitudinal, consistent stressor (Fig. 2). Although there was no significant interaction between religiosity and sex on depression, we did observe a significant sex difference on religious attendance. Specifically, men were more frequent religious attenders. We observed a significant difference between the population proportions in each religious attendance group and ethnicity. These ethnicity-based preferences could result from a difference in preferred religious rites, such as independent spiritual practices and community-based religious services. By performing the sensitivity analysis, our results are shown to be valid across ethnic groups. Only South/Southeast Asians had an inverse response compared to all other ethnic groups. The South/Southeast Asian population also had the lowest mean change in PHQ–9 score, indicating that these individuals are less susceptible to the typical onset of depression during internship (Fig. 3). In our study, African Americans were more likely than White interns to attend religious services. In contrast, those of East Asian ancestry are less likely to attend religious services. Notably, the Latino population seems to exhibit a strong protective response as the result of religious attendance; however, the significance of this result is confounded by small sample size (N, Latino: 210, N, Latino, more than once a week: 2). Overall, we found that for the majority of the ethnicities present in the studied intern population, religious attendance served as a resilience factor against an increase in PHQ–9 scores, and consequently depressive symptoms.

Identifying religious attendance as a protective factor against depressive symptom onset under stress in the IHS cohort is critical to understanding the multifaceted mental health state of interns undergoing this stressful period. While religion is often considered a private topic, making supervisors and instructors aware of this potential effect may allow them to respond with more understanding when circumstances involving religious attendance arise. Ultimately, while actionable interventions are not feasible or ethical concerning religious attendance, discovering the protective mechanism of religious attendance at the onset of severe stress is another key piece toward a comprehensive understanding of stress, resilience factors, and their impact on mental health.

Our study has several limitations. Although our results were consistent across most ethnic groups, a large proportion (59.0 %) of our sample was White. Further studies should be conducted in more diverse populations. Religious attendance was self-reported, and consequently subject to biases. Furthermore, our study focused on religious attendance, but did not stratify by religious affiliation, nor did we specifically measure the amount of religious attendance during the stressful period of life. We also only assessed overall religious attendance, which is not a complete measure of religiosity. Therefore, we were not able to differentiate people who are more religious but have limited access to religious service and those who are less religious. We also cannot distinguish whether religious attendance is protective because those with high practice experience more spirituality, because religious attendance offers social support and connectivity, or because of other factors such as having a purpose in life (Alimujiang et al., 2019; Wood & Joseph, 2010). However, it is notable that more frequent religious attendance has been found to be related to lower risk of deaths from despair – deaths related to drugs, alcohol, and suicide – in medical health professionals (Chen et al., 2020). A recent study indicates a positive causal effect of religious attendance in a heterogeneous population, even after adjusting for multiple confounders (Aksoy et al., 2022). Given that the same study did not identify a similar effect from the strength of religious belief, the authors suggest that the positive effect on mental health outcomes is potentially driven by social support and reduced loneliness. More work is needed to derive how the strength of religious belief might impact the mental health of a population facing stress, as represented by the IHS cohorts.

## 5. Conclusion

In conclusion, we found religious attendance to be protective against a rise in depressive symptoms in response to work stress among the high-risk population of medical interns. Furthermore, we found evidence supporting the notion of religious attendance functioning as a two-fold resilience factor, as it lessened the initial impact of the stressor, as well as had a continuous impact longitudinally. Identifying and understanding how resilience factors behave during stress is key to increasing our understanding of depression.

## Figures and Tables

**Table 1:** Sample demographic characteristics (*N* = 5482).

Sample characteristic	N	%

Sex		
Female	2954	53.9
Male	2528	46.1

Sample characteristic	Mean	SD

Age	27.5	2.8
Neuroticism	23.0	8.8
PHQ-9 at Baseline	2.6	3.0
Early Family Environment	13.0	9.6

**Table 2:** Comparing religious attendance between sexes and ethnicities^a^.

Population comparisons	t-Statistic	*p*-Value	Cohen’s D	Mean religious attendance of men	Mean religious attendance of women

Male, female	3.25 (df = 5221.1)	0.001	0.09	2.7	2.5

Population comparisons	t-Statistic	*p*-Value	Cohen’s D	Mean religious attendance of reference population (white)	Mean religious attendance of non-reference population

White, African American	−9.66 (df = 276.98)	<0.001	0.66	2.58	3.51
White, Arab	−2.51 (df = 93.97)	0.01	0.30	2.58	3.00
White, Southeast Asian	−4.30 (df = 832.28)	<0.001	0.18	2.58	2.83
White, East Asian	6.69 (df = 676.13)	<0.001	0.32	2.58	2.12
White, Latino	1.08 (df = 242.64)	0.28	0.07	2.58	2.48
White, Other	3.17 (df = 892.38)	0.002	0.14	2.58	2.38

a Results calculated using Welch’s t-test. Effect size calculated using Cohen’s *d*.

**Table 3:** The effect of religious attendance on PHQ-9 change and baseline PHQ-9.

Outcome	PHQ-9 change		Baseline PHQ-9	
	Effect Size	p-Value	Effect Size	p-Value
**Religiosity Group**	−0.10	.002	0.01	.322
Neuroticism	0.10	< .001	0.05	< .001
Absence of Prior Depression	−1.0	< .001	−0.19	< .001
Female Sex	0.15	.090	−0.08	.001
Work Hours	0.04	< .001		
Perceived Medical Error	0.74	< .001		
Baseline PHQ-9	−1.38	< .001		
Average Hours Slept	−0.58	< .001		
Early Family Environment			0.01	< .001
Stressful Life Event at Baseline			0.18	< .001
Year 2018	−0.05	.604	0.04	.127
Year 2019	−0.32	.005	0.08	.009
	Adjusted R2	p-Value	Adjusted R2	p-Value
Overall Model	.211	< .001	.25	< .001

Cells are grey where a variable was not used in that model.

**Table 4:** Mean PHQ-9 change by religious attendance group.

Religious attendance	N	Mean PHQ-9 Change [SD]

Never	1712	3.5 [3.7]
Once a year or less	1023	3.5 [3.6]
A few times a year	1451	3.4 [3.4]
A few times a month	572	3.2 [3.6]
Once a week	585	3.1 [3.6]
More than once a week	139	2.2 [3.1]

## Data Availability

The de-identified data from Intern Health Study that support the findings described here are available from the corresponding author upon reasonable request.
